# The Cyclin-Dependent Kinase 5 Inhibitor Peptide Inhibits Herpes Simplex Virus Type 1 Replication

**DOI:** 10.1038/s41598-018-37989-3

**Published:** 2019-02-04

**Authors:** Adrian Man, Mark Slevin, Eugen Petcu, Cornel Fraefel

**Affiliations:** 1 Institute of Virology, University of Zurich, Zurich, Switzerland; 2 Department of Microbiology, University of Medicine and Pharmacy of Tîrgu Mureș, Târgu Mureș, Romania; 3 University of Medicine and Pharmacy of Tîrgu Mureș, Târgu Mureș, Romania; 4 School of Healthcare Science, Manchester Metropolitan University, Manchester, UK; 5 Griffith University, Gold Coast, Brisbane, Australia

## Abstract

In order to evaluate the influence of CDK5 inhibitory peptide (CIP) on *Human alphaherpesvirus 1* (HSV-1) replication, we constructed two recombinant adeno-associated-virus 2 (rAAV2) vectors encoding CIP fused with cyan-fluorescent-protein (CFP), with or without nuclear localization signal. A third vector encoding non-fused CIP and CFP was also constructed. HeLa and HEK 293T cells were infected with the rAAV-CIP vectors at multiplicity of infection (MOI) of 5000, in the absence or presence of a recombinant HSV-1 that encodes a yellow-fluorescent-protein (rHSV48Y; MOI = 1). Cells co-infected with rHSV48Y and rAAV vectors that did not express the CIP gene (rAAV-CFP-Neo) served as controls. At 24 h after infection, the effect of CIP on rHSV48Y replication was assessed by PCR, qRT-PCR, Western-blot, flow-cytometry, epifluorescence and confocal microscopy. We show that in cultures co-infected with rAAV-CFP-Neo, 27% of the CFP-positive cells present rHSV48Y replication compartments. By contrast, in cultures co-infected with CIP-encoding rAAV2 vectors and rHSV48Y only 6–20% of the cells positive for CIP showed rHSV48Y replication compartments, depending on the CIP variant. Flow-cytometry showed that less than 40% of the rHSV48Y/rAAV-CIP, and more than 75% of rHSV48Y/rAAV-CFP-Neo co-infected cells were positive for both transgene products. The microscopy and flow-cytometry data support the hypothesis that CIP is inhibiting HSV-1 replication.

## Introduction

*Human alphaherpesvirus 1*, also known as herpes simplex virus type 1 (HSV-1), is a species in the genus *Simplexvirus*, family *Herpesviridae*, order *Herpesvirales*. It is one of the most common human pathogens, largely spread due to its oral to oral dissemination, with an estimated prevalence of 67%, an incidence of 118 million and a prevalence of 3.7 billion in 2012^1^. The herpetic lesions are located in mostly in perioral area, but ocular, skin mucous membrane lesions are also frequent. Severe complications such as recurrent infections, aseptic meningitis, keratitis, encephalitis, neonatal herpes, visceral involvement, including HSV-1 pneumonia can occur, especially in immune-compromised patients. Common therapeutic options include acyclic nucleoside analogues (Acyclovir, Famcyclovir) that act as viral DNA polymerase inhibitors. Acyclovir resistant strains are described since 1980s^2^ and is still a problem^3^, so alternative treatment is required in these cases, but with higher costs and toxicity risks^4^. Prevention against HSV-1 infection is not yet possible in humans, though experimental vaccines are developed and tested on animals^5^. New therapeutic and prophylactic measures against HSV-1 have to be developed, such as gene-therapy using viral vectors. Recombinant Adeno-associated virus 2 (rAAV2) vectors, in contrast with the wild-type Adeno-associated viruses, were found to be safe and effective in preclinical and clinical settings, as they cannot replicate, do not contain any virulence genes and do not integrate into host genomes. Instead, the encoded transgenes can form circular concatemers that persist as episomes in the nucleus of the infected cells. In addition, rAAV2 vectors present a broad tissue tropism, which makes them usable in multiple pathologies. These vectors can be easily engineered to include a DNA sequence of interest of up to 5 kb^6^.

## CDK5 and Its Activators and Suppressors

CDK5 is a 32 kDa protein composed of 292 amino-acids with ubiquitous expression, but with proline-directed serine/threonine kinase activity mainly in post-mitotic neurons. CDK5 is present also in other cell types, but its activity in neurons is directly linked with the high level of p35 and p39^7^. It is an atypical kinase, as compared to CDK1-4 and -6 that regulate cell cycle progression, CDK5 acts as a regulatory kinase in several post-mitotic processes such as neuronal activity, neuronal migration during development and neurite outgrowth^8^. Compared to the other cyclin-dependent kinases, CDK5 does not need to be phosphorylated in order to express its kinase activity^9^. Though CDK5 is present in higher amount in the cytoplasm, its kinase activity is higher in the nucleus, probably due to the higher amounts of nuclear p35. One of the main activities is the Retinoblastoma (Rb) phosphorylation at several C-terminus sites (780, 788, 795, 807, 811, 821, 826) as proved by mass spectrometry, behaving similarly to Cdk2/Cyclin E (phospho-specific antibodies of Rb are limited to Ser780, Ser795 and Ser807/811). Dephosphorylated Rb protein binds to E2F1 and turns it “OFF,” while phosphorylated Rb protein dissociates from E2F1 and turns it “ON.” In normal condition, CDK5 does not affect the activity of E2F1 even though it phosphorylates Rb; more than this, CDK5 suppresses the neuronal cell cycle by disrupting the E2F1-DP1 complex and arrest the cell cycle in G1 state. Instead, the overexpression of CDK5 increases the Rb phosphorylation, followed by the initiation of E2F1 activity, stimulating the neuronal cell cycle, leading to neuron apoptosis^10,11^. CDK5 is activated by p35 (CDK5R1) and p39 (CDK5R2 – a 367 aa isoform of p35), hyperactivated by p25, and inhibited by Cyclin-dependent kinase inhibitor 1 (p21), CIP, p5 or several chemical compounds (roscovitine, resveratrol, AT-7519 and olomoucine)^12^.

The role of CDK5 in other tissues than neurons remains poorly described. It was shown that CDK5 may have a role in tumorogenesis, or that the inhibition of CDK5 may have antitumoral activity by altering the expression of several cell-cycle related proteins^13,14^.

p35 (CDK5R1) is a 35 kDa protein expressed mostly in CNS, in post-mitotic neurons, with regulatory function (activates CDK5) and present high specificity for CDK5, but not for other kinases as CDK2, CDK3, CDK4, DCK6 or CDC2^15,16^. It has a short half-life of 20–30 minutes. p35 is produced after the cleavage of the initiator methionine from Cyclin-dependent kinase 5 activator 1, which is encoded by CDK5R1 on 17q11.2^17,18^. It is non-soluble, being associated with membrane/cytoskeleton. The phosphorylation of p35 by CDK5 is a signal for ubiquitination/degradation. p35 is found mostly in phosphorylated state in fetal brain, while the non-phosphorylated state is found mostly in adult brain^7^. In addition, P21/WAF1 is an inhibitor of cyclin-dependant kinases 2,3,4,6, with a lower activity on CDK5^19^. Its binding to cyclin/CDK complexes leads to the inhibition of Rb protein phosphorylation.

**Figure 1:**
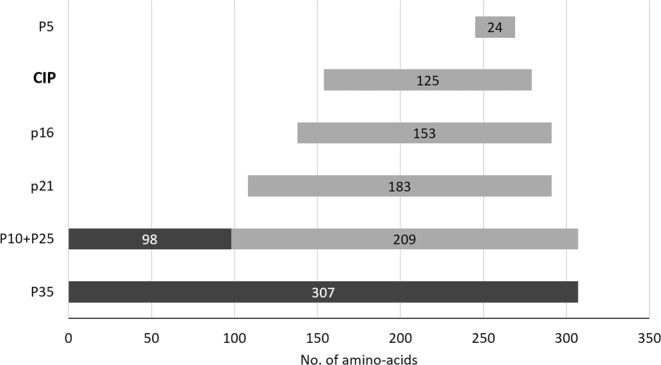
Splice products of p35. P25 is a CDK5 hyperactivator, while p21, p16, CIP and p5 are CDK5 inhibitors.

CDK5 inhibitory peptide (CIP) is a 125 aa (154–279), 12.5 KDa peptide that is generated after the C- and N-terminal truncation of p35 (Supplemental Fig. 1), with a high affinity for CDK5 and a longer half-life^20^. The splice products of p35 are presented in Fig. 1.

We present a new finding of HSV-1 inhibition by a small Cyclin-dependent kinase 5 (CDK5) inhibitory peptide (CIP). Our hypothesis is that CIP alters one or several cell-cycle related functions, leading to a reduction in the proper expression of key viral proteins, and concluded in the inhibition of HSV-1 replication. It has been previously shown that CDK5 activity has great importance in modulating neuronal cell-cycle, but it is less known whether it has any effects in normal cells or over viral replication.

Our goal was to assess HSV-1 replication in cells coinfected with Adeno-Associated Viral vectors that express CIP as an inhibitor of CDK5.

## Results

### CIP-encoding viral vectors

**Figure 2:**
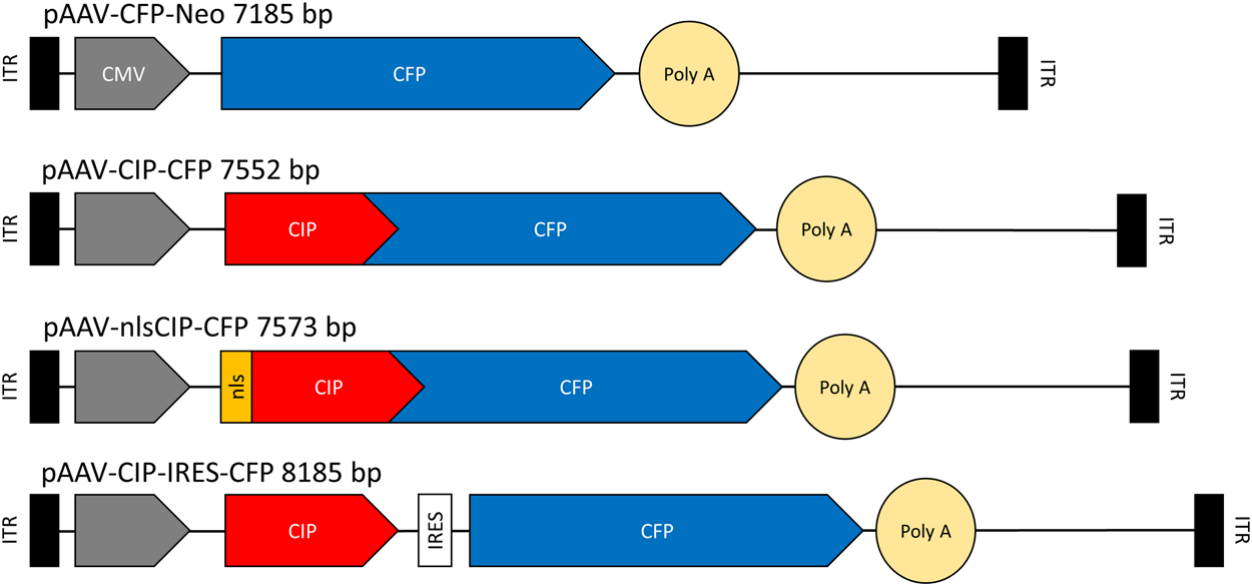
Schematics of the inserts of rAAV-CIP constructs.

To investigate the effect of CIP overexpression on HSV-1 replication, we constructed recombinant AAV2 vectors that encode CIP alone or fused with cyan fluorescent protein, with or without nuclear localization signal and CMV promoter (Fig. 2). The titer of rAAV2 amplicons was assessed by qPCR and presented values between 8 * 10^9^ to 3.4 * 10^10^ DNA copies/ml, depending on the type of construct.

### Transgene expression

**Figure 3:**
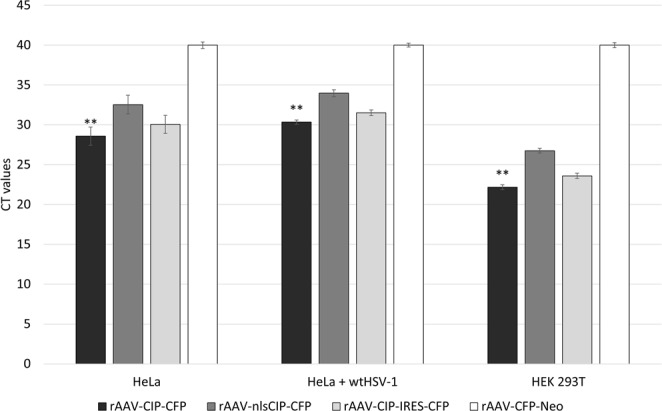
Data showing the expression of CIP in cells infected with rAAV2-CIP vectors (CT values). CIP is better expressed by rAAV-CIP-CFP than by rAAV-nlsCIP-CFP (p < 0.05), and better in HEK 293T cells regardless of the vector type (p < 0.05). The CT values for mock infected cells and for cells infected with rAAV-CFP-Neo were higher than 39 or not detectable. The data represent a triplicate experiment and are shown as average CT values ± standard deviation for each detection. HeLa cells coinfected with wtHSV-1 and rAAV2-CIP vectors were used in order to check if HSV-1 (as AAV2 helper virus) is influencing CIP expression. **P < 0.05 (One-way ANOVA for comparison of mean values with Bonferroni post-test) when comparing CIP expression for the three different viral vectors.

To test transgene expression, HeLa cells were infected with the rAAV-CIP-CFP, rAAV-nlsCIP-CFP or rAAV-CIP-IRES-CFP at a MOI of 5000. Reverse-transcription PCR (RT-PCR) gene expression for CIP showed good amplification, confirming the CIP expression. Quantitative reverse-transcription PCR (qRT-PCR) further confirmed CIP expression in HeLa cells, in HeLa cells coinfected with wtHSV-1 (as AAV2 helper virus) and in HEK 293T cells (that present Adenoviral E1A and E1B helper genes in their chromosome, SV40 Large T-antigen and which allows better AAV transgene expression) (Fig. 3; Supplemental Fig. 2). Forcing the nucleus internalization of CIP by using the nuclear-localization-site coding sequence lead to a lower expression of CIP. Internal ribosome entry site (IRES) is a cis-acting element that recruit the 40s ribosomal subunits to an initiator codon in the mRNA. Thus, the two encoded proteins (CIP and CFP) will be expressed independently. Nevertheless, in our rAAV-CIP-IRES-CFP construct, the fist gene (CIP) was expressed in a lower amount than by rAAV-CIP-CFP that does not include IRES. IRES system is not fully understood, the literature describing usually a lower expression of the second gene in IRES constructs^21,22^. Further experiments would have to be performed in order to assess the independent co-expression of CIP and CFP, and why the CIP expression is lower than in the CFP-fused version, especially when talking about experiments which involve alterations of the cell cycle.

### Assessment of HSV-1 replication

We used a recombinant HSV-1 encoding VP16 fused with the enhanced yellow fluorescent protein (rHSV48Y) to test its replication in presence of rAAV-CIP vectors that express CFP.

From the whole HeLa cells lysates co-infected with rAAV-CIP and rHSV48Y, we found no difference in the HSV VP16 expression (Supplemental Fig. 3). Also, there were no significant differences in HSV DNA level from whole cell lysates. This can be explained by the intensive replication of HSV in the cells that were not infected by our rAAV-CIP vectors, so high amounts of VP16 and HSV-1 DNA were present in whole cell lysates.

**Figure 4:**
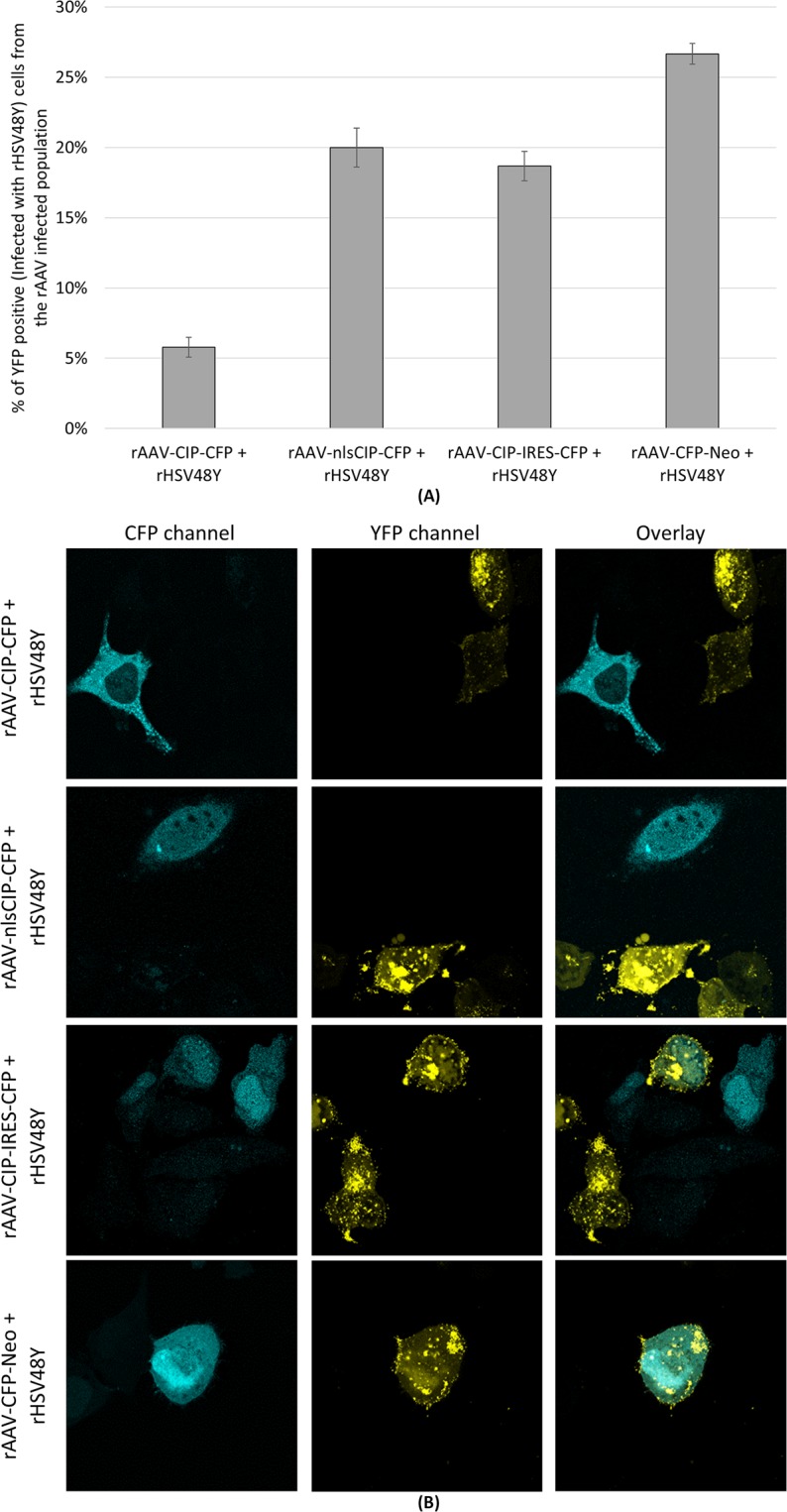
(**A**) Graph showing the proportion of HeLa cells infected with rHSV48Y from the population of cells expressing CIP, represented by mean values of the field counts (n = 15 fields, counted in triplicate); the error bars represent the standard deviation of the counts. (**B**) Suggestive images from confocal microscopy following the HeLa cells coinfection with rAAV-CIP vectors and rHSV48Y (recombinant HSV-1 that encodes yellow-fluorescent-protein). Cells infected with rAAV that do not express CIP are coinfected with rHSV48Y.

By following and counting individual cells by fluorescence and confocal microscopy, we found that in cultures co-infected with rAAV-CFP-Neo, 26.7% ± 0.7% of the CFP-positive cells presented rHSV48Y replication compartments (VP16 fused with YFP). By contrast, in cultures co-infected with CIP-encoding rAAV2 vectors and rHSV48Y, less than 20% of the cells positive for CIP showed rHSV48Y replication compartments, depending on the CIP variant (Fig. 4A,B; Supplemental Fig. 4).

**Figure 5:**
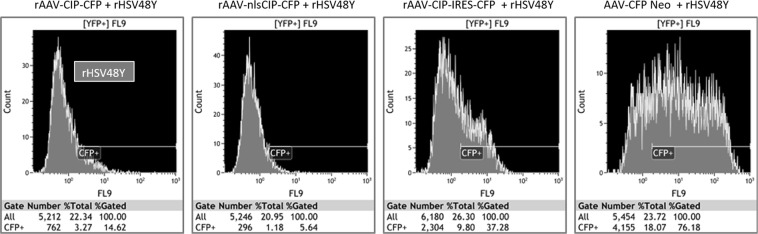
CFP-positive HeLa cells from the rHSV48Y infected population. Flow-cytometry showed that less than 40% of the rHSV48Y/rAAV-CIP, and more than 75% of rHSV48Y/rAAV-CFP-Neo co-infected HeLa cells were positive for both transgenes products.

To further confirm if the replication of HSV-1 is affected by CIP, we assessed the rate of HSV-1 coinfection on CIP presenting cells by flow-cytometry. Notably, from all rHSV48Y infected cells (gated on YFP positive channel), less than 40% were CIP-positive (gated on CFP channel from rAAV vectors), indicating that HSV is replicating preferentially in cells that does not express CIP (Fig. 5). The most efficient rAAV-CIP variant in inhibiting HSV-1 replication was rAAV-nlsCIP-CFP, as rHSV48Y managed to coinfect only 5.64% of cells with nuclear localization of CIP. Conversely, more than 75% of rHSV48Y/rAAV-CFP-Neo co-infected cells were positive for both transgene products, indicating that in cells not expressing CIP but infected with AAV2, HSV-1 can replicate.

## Discussion

### CDK5 regulation and cell cycle

CDK5 is a serine-threonine kinase with great importance in neuronal development and survival, aside other cyclins with similar homology; its effects and implication are less known on non-neuronal cells, where other Cyclin-dependent kinase and cyclins such as CDK1, CDK2, CDK4, CDK5, Cdc2, Cyclin D1, Cyclin E are more active^23,24^.

CDK5 activity is mainly regulated by p35, which acts as a physiological activator, especially in neurons, where it exerts a protective role, including against viral induced apoptosis^25^. p35 cleavage products present different effects: P25 is an hyperactivator, whereas P21, CDK5 Inhibitory Peptide (CIP) and P5 are inhibitors of CDK5 activity in neurons^26,27^. CIP is a 125 aminoacid peptide, generated after a further cleavage of P25, with a selective inhibitory effect on p25/CDK5, thus preventing CDK5 hyperactivation *in vivo*, but without affecting the normal p35/CDK5 function^26^.

Activated CDK5 (CDK5/p25) increase the expression of p53 (most likely by phosphorylating Ser33 and/or Ser315), and activated p52 increase the expression of p21. CDK5 inhibitors (roscovitine) inhibits p53 phosphorylation by CDK5, possibly by interfering with the nuclear translocation of CDK5 from cytoplasm^28^.

### Cell cycle and replication of HSV-1

HSV-1 can efficiently replicate in actively dividing cells; unlike other viruses, it can also replicate (though in less amount) in growth-arrested cells. Nevertheless, HSV-1 replication is dependent on several cell cycle related factors. For example, pRb, E2F, p53, Cdk2, PCNA, RPA, are all accumulating in late G1 and S phase of cell cycle and seem to play important roles in HSV-1 replication^29^. It was already shown that p35, aside CDK5, plays a key role in HSV-1 replication *in vivo*^25^. Our previous experiments also suggest that part of the mentioned factors (pRB, E2F) are down-regulated under effect of CIP^14^, so we can suspect that HSV-1 inhibition by CIP is related to the alteration of CDK5/p35 cellular functions.

It is known that HSV-1 presents a series of mechanisms that make use of CDK5 and p35 and even increase their levels in order to thrive in neurons. Neurons are quiescent cells when they reach maturity (blocked in G0 differentiation phase), and they undergo apoptosis in response to cell cycle reactivation^30^. The oxidative stress of HSV-1 infected cells induce Egr-1 activation, followed by induction of p35 and cytoplasmic localization of CDK-5, prolonging the survival of infected neurons by preventing the cell-cycle reentry^31^.

HSV-1 replication is inhibited by several cell cycle inhibitors such as Roscovitine or Olomucine. Both block VP16-dependent IE gene expression, but also block the cell-cycle progression in late G1/S phase by altering CDKs activity^29^.

Zhou *et al*. have shown that HSV replication is inhibited due the disruption of p53-p21 pathway, where the knockdown of p53 or p21 resulted in a significant reduction of HSV production^32^. Activation of p53 leads to up-regulation of p21, a CDK5 inhibitor similar to CIP.

HSV-1 requires immediate early proteins such as ICP0 for its replication, as they are important transactivators of HSV early and late gene expression. Previous studies showed that ICP0 is not found in the nucleus of the infected neurons, despite expression of ICP0 mRNA^33^. Without ICP0, major viral proteins (VP16) will not be expressed, leading to inhibition of HSV-1 replication and progression into latency in infected neurons^34^.

All these data suggest that HSV-1 replication needs functional CDKs. As our rAAV-CIP vectors successfully expressed CIP into the infected cells, its CDK5 inhibitory effect was responsible for the inhibition of HSV-1 replication.

The microscopy and flow-cytometry data support the hypothesis that CIP is inhibiting HSV-1 replication. Most of the CIP positive cells did not present visible HSV-1 replication sites. Most of the HSV-1 infected cells were not co-infected with rAAV-CIP-CFP, but co-infected with rAAV-CFP-Neo. Nuclear localization of CIP is associated with the strongest HSV inhibition.

## Material and Methods

### Cell lines

HeLa cells and human embryonic kidney cells (HEK 293T) were part of the collection of the University of Zurich, Department of Experimental Virology. The cells were thawed from −140 °C DMSO stocks. The cells were grown in high glucose-containing Dulbecco’s Modified Eagle’s Medium (DMEM) with 4500 mg/L glucose containing 10% FBS, 100 U/ml penicillin, 100 μg/ml streptomycin and 100 μg/ml amphotericin B at 37 °C in 5% CO_2_ atmosphere. Cells were revitalized in T25 flasks and when 70–80% of confluence was reached, the cells were subcultured once more before using them further.

### Plasmids and rAAV vectors

Plasmids containing recombinant AAV2 genomes pAAV-CIP-CFP, pAAV-nlsCIP-CFP (CIP with and without nuclear localization signal NLS, fused with CFP) and pAAV-CIP-IRES-CFP (CIP not fused with CFP) were constructed by inserting CIP coding sequence in the pAAV-CFP-Neo. The pAAV-CFP-Neo is a plasmid that include a backbone consisting in CMV promoter, CFP and a poly(A) site, all inserted between two ITRs (inverted terminal repeats).

### CIP and nlsCIP sequence design

A DNA fragment containing the CIP coding sequence was amplified by PCR from plasmid pLV-CIP (a kind gift from Prof. Sashi Kesavapany, National University of Singapore) using the following conditions: 1 min 98 °C, 35x amplification steps (10 sec. 98 °C, 15 sec. 55 °C, 30 sec. 72 °C), final elongation 10 min 72 °C. Custom designed Forward primers were used for CIP PCR: the Forward primer contained AfeI restriction site (AGC ⇅ GCT), Kozak consensus sequence (5′ CATGTGC 3′), with/without nuclear localization signal NLS (5′ CCTAAGAAGAAGCGTAAGGTG 3′) and first 18 nucleotides from CIP coding sequence (5′ CTGGGTGAGTTTCTCTGC 3′). The reverse primer contained AfeI restriction site and the last 21 nucleotides complementary to the CIP coding sequence (5′ GGCATTTATCTGCAGCATCTT 3′). The primers’ length was adjusted in order to be in line with the open reading frame of CFP.

### pAAV-CIP-CFP and pAAV-nlsCIP-CFP

Digestion of pAAV-CFP-Neo was performed with AfeI (New England Biolabs, Ipswich, MA) for 2 h at 37 °C, followed by dephosphorylation of the 5′ and 3′ ends with Alkaline phosphatase (New England Biolabs, Ipswich, MA) for 20 minutes at 37 °C. Inactivation of the enzymes was performed for 5 minutes at 65 °C. The AfeI restriction site in pAAV-CFP-Neo is located at the junction between the CMV promoter and CFP-coding gene. CIP and nls-CIP PCR products were digested with AfeI and demethylated (DpnI) for 2 h at 37 °C and then purified. The 7185 bp pAAV-CFP-Neo linearized fragment was ligated with the 385 bp CIP fragment or with the 406 bp nlsCIP fragment using T4 DNA Ligase (New England Biolabs, Ipswich, MA) for 14 hours at 16 °C. In order to create a functional CIP-CFP fused protein, the in-frame “Stop” codon TAG present in the fusion coding area was removed by SbfI/BamHI double digestion, followed by blunting of the ends (T4 DNA Polymerase) and ligation (T4 DNA Ligase).

### pAAV-CIP-IRES-CFP

Plasmid pAAV-CIP-IRES-CFP was constructed by inserting an IRES coding sequence between CIP and CFP in pAAV-CIP-CFP. The IRES coding sequence was amplified by PCR from pHSV-IRES1-eGFP (collection of the University of Zurich, Department of Experimental Virology) using custom designed primers containing XhoI and SalI restriction sites (Forward primer: TAACTCGAGAAAAGCTTTTAAAACAGC; Reverse primer: TAAGTCGACCAATCCAATTCGCT). Both the IRES PCR products and pAAV-CIP-CFP were treated with XhoI and SalI. The 642 bp fragment containing the IRES coding sequences was ligated with the 7539 bp pAAV-CIP-CFP fragment using T4 DNA Ligase (New England Biolabs, Ipswich, MA) for 14 hours at 16 °C.

### Transformation of DH-10B competent cells

DH-10B competent cells were transformed using heat-shock method. AMP resistant bacterial colonies grown on Luria-Bertani Agar supplemented with ampicillin 100 μg/ml were processed for plasmid DNA extraction (Qiagen Plasmid Mini Kit, Qiagen, Hilden, Germany); pAAV-CIP-CFP and pAAV-nlsCIP-CFP were checked for the proper orientation of the insert using SacI digestion and electrophoresis of digested products. All plasmids were sequenced to confirm the presence of inserted CIP, nlsCIP or IRES coding sequences. For each successful clone, 7% DMSO bacterial stocks were prepared.

### Cell culture, transient transfection and virus purification

HEK 293T cells were grown on 20 cm corning dishes containing DMEM medium supplemented with 10% FBS and 1% Antibiotic-Antimycotic. Recombinant AAV2 stocks were produced by transient transfection of HEK 293T cells, using the helper plasmid pDG (PlasmidFactory, Heidelberg, Germany) which contains all helper and packaging functions required for rAAV production (rep2/cap2), and either pAAV-CIP-CFP, pAAV-nlsCIP-CFP, or pAAV-CIP-IRES-CFP. Virus stocks were purified, following the general guidelines described by Shin *et al*.^35^ and their titer was assessed by qPCR using specific CFP primers. rAAV-CFP that does not encode CIP was similarly produced using pDG and pAAV-CFP, isolated and used as negative control in all experiments.

### Herpes viruses

Wild-type HSV-1 (wtHSV-1) and recombinant HSV-1 that encodes a yellow-fluorescent-protein (YFP) fused with VP16 (rHSV48Y) were thawed from −80 °C unconcentrated vector stocks from the collection of the University of Zurich, Department of Experimental Virology. Both types of viruses were successfully used in previous research^36^, and for rHSV48Y it was shown that the fusion of EYFP to VP16 does not significantly affect the viral titer compared to wtHSV-1.

### Validation of CIP and CFP expression in infected cells

HeLa and HEK 293T cells were infected with rAAV vectors, MOI 5000. After 24 hours of incubation, RNA was extracted using TRIzol reagent (ThermoFisher Scientific) and Direct-zol™ RNA MiniPrep Kit (ZymoResearch, USA), considering the fact that the expression levels derived from the total RNA fraction correlates with the amount of mRNAs present in a cell population^37^. cDNA was prepared using GoScript™ Reverse Transcription System Protocol (Promega, USA). PCR, electrophoresis of amplified products and qRT-PCR was performed using CIP primers (Forward: 5′ CTGGGTGAGTTTCTCTGC 3′; Reverse: 5′ GGCATTTATCTGCAGCATCTT 3′). CFP expression was assessed both by qRT-PCR (Forward primer: 5′ CCGAGGTGAAGTTCGAGG 3′; Reverse primer: 5′ GCCGTTCTTCTGCTTGTC 3′) and Western-blot using anti-CFP antibodies.

### Assessment of HSV-1 replication

Replication of HSV in presence of CIP gene was assessed by co-infecting HeLa cells with rAAV vectors multiplicity of infection (MOI) of 5000 with rHSV48Y (MOI = 1) or with wtHSV-1 (MOI 1). Cells co-infected with HSV-1 and rAAV vectors that do not express the CIP gene (rAAV-CFP-Neo) served as controls. After 24 hours of incubation, virus infection/replication was evaluated by Western blot, PCR, epifluorescence and confocal microscopy and flow-cytometry.

### Western blot analysis

Infected HeLa cells were harvested after 24 hours of incubation and lysed in Laemmli sample buffer (Bio-Rad, Hercules, CA) containing 5% 2-Mercaptoethanol. Proteins were resolved using sodium dodecyl sulfate polyacrylamide gel electrophoresis (SDS-PAGE) and transferred to PVDF membranes (Millipore, Billerica, MA, USA). After blocking for 1 h with 5% non-fat-dry-milk solution in PBS-T (phosphate buffered saline supplemented with 0.3% Tween 20), the specific rHSV48Y proteins from the membrane were probed overnight with specific rabbit monoclonal antibodies against VP16 (diluted 1:1,000 in PBS-T, kindly provided by A. Minson and H. Browne, University of Cambridge, United Kingdom). The membranes were washed three times with PBS-T and incubated with secondary goat anti-rabbit peroxidase-conjugated antibodies (1:10,000 in PBS-T; Sigma-Aldrich, Buchs, Switzerland) for 1 h at room temperature. Target proteins were detected by the enhanced chemiluminescence detection system (WesternBright ECL Spray, Advansta Inc., Menlo Park, CA, USA). Actin detection was used as loading control for all blots. A molecular weight marker (PageRuler Plus Prestained Protein Ladder; ThermoFisher Scientific) was used to determine the sizes of the protein bands.

### qPCR for HSV-1 DNA

The number of HSV-1 genome containing particles in presence of CIP was assessed by qPCR. HeLa cells were coinfected with rAAV-CIP vectors and wtHSV-1. After 24 hours, whole DNA was extracted from the coinfected cells (DNeasy Blood & Tissue Kit, Qiagen, Hilden, Germany). HSV-1 DNA was quantified by qPCR using specific primers for the HSV-1 UL35 gene (Forward: GTCTTGGCCACCAATAACTC; Reverse: GGGTAAACGTGTTGTTTGCG) and the results were normalized against GAPDH and rAAV-CFP-Neo/wtHSV-1, using ΔΔCT method.

### Microscopy

For further evaluation of HSV-1 replication, a microscopical screening was performed from a rAAV/rHSV48Y coinfected cell culture. For this, for each rAAV vector, fifteen random field-of-view screenshots were captured on CFP and YFP channels using an epifluorescence microscope, with a magnification power of 40x. The images were merged, and all the cells were noted in a spreadsheet (CFP-positive, YFP-positive, respectively coinfected cells). The cell counting was performed in triplicate. To assess the possible HSV-1 inhibition by CIP, the CFP-positive cells were followed, and among them, the percentage of cells that present yellow HSV-1 replication compartments were noted. Leica TCS SP2 (Leica Microsystems, Wetzlar, Germany) confocal microscope was also used for checking rAAV-CIP/ rHSV48Y colocalization.

### Flow-cytometry

Complementary to microscopy, Beckman Coulter Gallios Flow Cytometer was used in order to assess a larger number of cells with greater accuracy, but in this case we followed the number of CIP-positive cells among the HSV-1 infected cells (by gating the YFP positive cells and then counting the CFP positive cells). HeLa cells were used as negative controls to set the CFP + gate.

All data generated or analyzed during this study are included in this published article.

### Statistical data

All numerical data from flow-cytometry cell count, microscopy cell count and the CT values from PCR were inserted in spreadsheet software and statistically processed. One-way ANOVA test with Bonferroni post-test was used to assess the significance of CIP expression by qPCR, by comparison of mean CT values.

## Supplementary information

Supplementary material
