# Supplementary material for: The Cyclin-Dependent Kinase 5 Inhibitor Peptide Inhibits Herpes Simplex Virus Type 1 Replication
Source: Sci Rep. 2019 Feb 4;9:1260. doi: 10.1038/s41598-018-37989-3 (PMC6362106; doi:10.1038/s41598-018-37989-3)
Supplement: Supplementary file 1 — Supplementary material [file 41598_2018_37989_MOESM1_ESM.pdf]

The Cyclin-Dependent Kinase 5 Inhibitor Peptide Inhibits Herpes Simplex Virus Type 1 Replication

Adrian Man [1,2]; Mark Slevin *[3,4], Eugen Petcu [5], Cornel Fraefel [1]

1. *Institute of Virology, University of Zurich, Zurich, Switzerland*
2. *Department of Microbiology, University of Medicine and Pharmacy of Tîrgu Mureș, Romania*
3. *University of Medicine and Pharmacy of Tîrgu Mureș, Romania*
4. *School of Healthcare Science, Manchester Metropolitan University, Manchester, UK*
5. *Griffith University, Gold Coast, Brisbane, Australia*

*Correspondence to: M.A.Slevin@mmu.ac.uk

```
        10         20         30         40         50
MGTVLSLSPS YRKATLFEDG AATVGHYTAV QNSKNAKDKN LKRHSIISVL
        60         70         80         90        100
PWKRIVAVSA KKKNSKKVQP NSSYQNNITH LNNENLKKSL SCANLSTFAQ
       110        120        130        140        150
PPPAQPPAPP ASQLSGSQTG GSSSVKKAPH PAVTSAGTPK RVIVQASTSE
       160        170        180        190        200
LLRCLGEFLC RRCYRLKHLS PTDPVLWLRS VDRSLLLQGW QDQGFITPAN
       210        220        230        240        250
VVFLYMLCRD VISSEVGSDH ELQAVLLTCL YLSYSYMGNE ISYPLKPFLV
       260        270        280        290        300
ESCKEAFWDR CLSVINLMSS KMLQINADPH YFTQVFSDLK NESGQEDKKR
       307
LLLGLDR
```

■ p10 ■ p25 ■ CIP

CIP gene sequence (direct strand +):

```
TGCCTGGGTGAGTTTCTCTGCCGCCGGTGCTACCGCCTGAAGCACCTGTCCCCCACGGACCCCGT
GCTCTGGCTGCGCAGCGTGGACCGCTCGCTGCTTCTGCAGGGCTGGCAGGACCAGGGCTTCATCA
CGCCGGCCAACGTGGTCTTCCTCTACATGCTCTGCAGGGATGTTATCTCCTCCGAGGTGGGCTCG
GATCACGAGCTCCAGGCCGTCCTGCTGACATGCCTGTACCTCTCCTACTCCTACATGGGCAACGA
GATCTCCTACCCGCTCAAGCCCTTCCTGGTGGAGAGCTGCAAGGAGGCCTTTTGGGACCGTTGCC
TCTCTGTCATCAACCTCATGAGCTCAAAGATGCTGCAGATAAATGCCGACCCAT
```

Supplemental Figure 1. CIP structure. CIP is a 125 aminoacid peptide with a molecular weight of 12.5 kDa.

(A) RT-PCR results

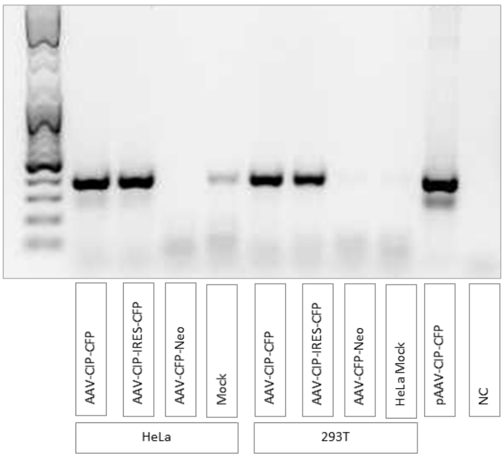

(B) qRT-PCR results

Cell lines	Replicate	CT values		
		rAAV-CIP-CFP	rAAV-nlsCIP-CFP	rAAV-CIP-IRES-CFP
HeLa	1	29.73	33.71	31.19
	2	27.43	31.38	28.94
	3	28.57	32.56	30.06
HeLa + wtHSV-1	1	30.59	34.09	31.67
	2	30.39	34.35	31.78
	3	30.06	33.50	31.12
293T	1	22.41	27.00	23.79
	2	22.30	26.83	23.79
	3	21.83	26.41	23.22

Supplemental Figure 2. Data showing PCR results for CIP expression by electrophoresis of the RT-PCR products (A) or by qRT-PCR CT values (B).

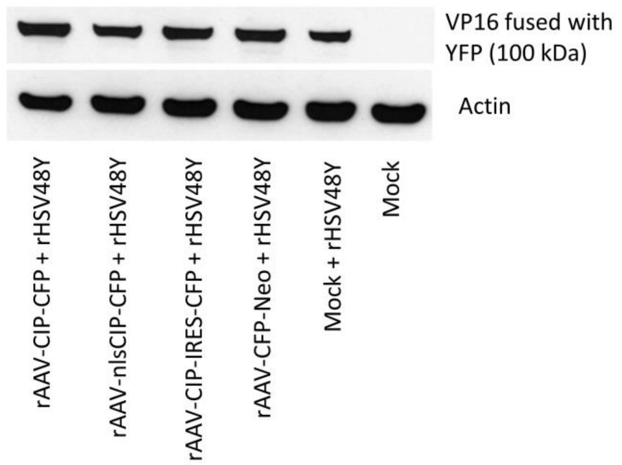

Supplemental Figure 3 (cropped image). Western-blot for HeLa cells co-infected with rAAV vectors and rHSV48Y.

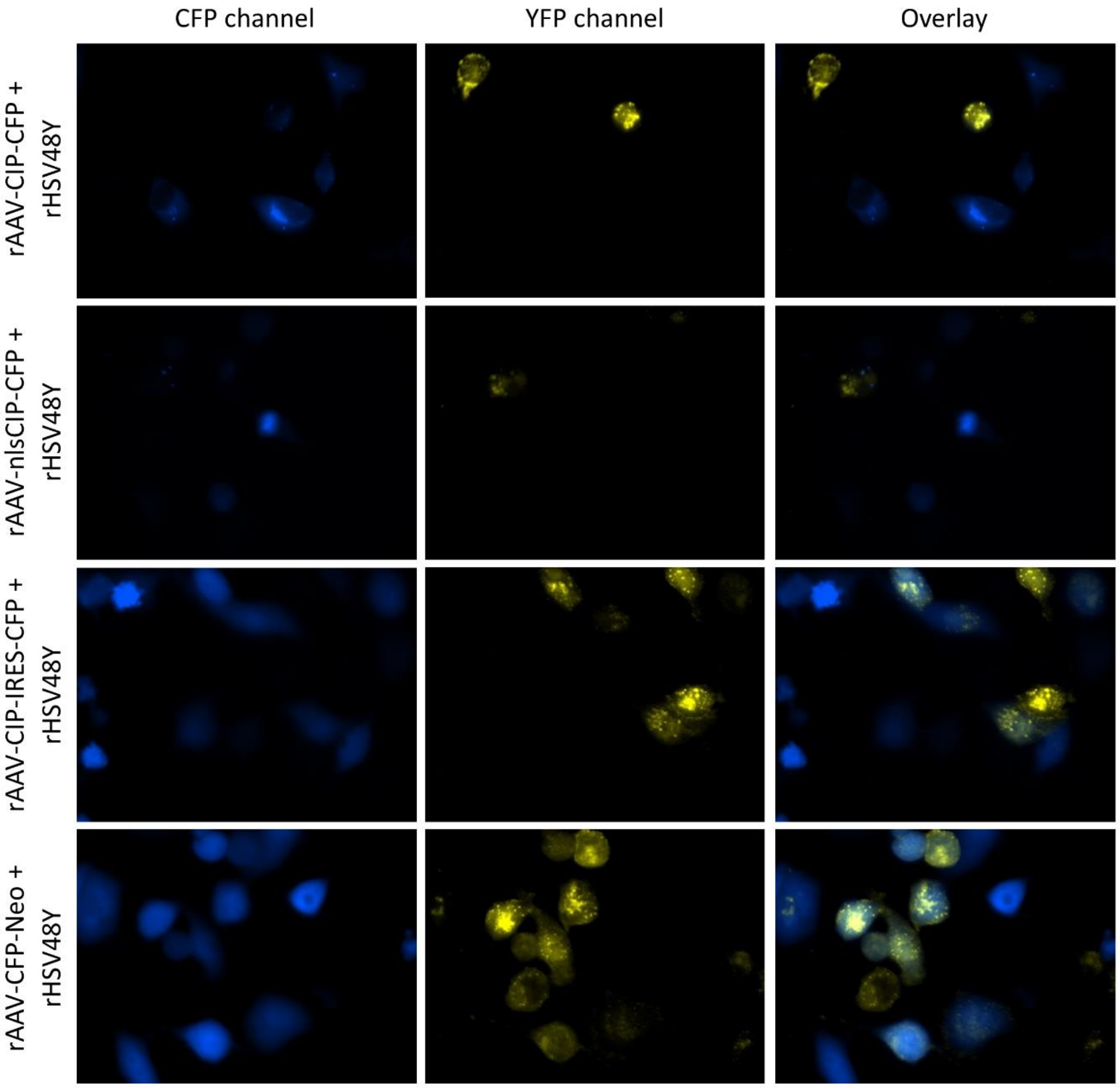

Supplemental Figure 4. Suggestive images from epifluorescence microscopy following the coinfection of rAAV-CIP vectors and rHSV48Y
